# Effect of anti-anxiety therapy on the prognosis of patients with atrial fibrillation and anxiety: a 2 × 2 factorial randomized controlled trial

**DOI:** 10.3389/fcvm.2026.1813078

**Published:** 2026-07-07

**Authors:** Zuoan Qin, Xuelin Lu, Ying Li, Liangqing Ge

**Affiliations:** 1 Department of Cardiology, Changde Hospital, Xiangya School of Medicine, Central South University (The First People’s Hospital of Changde City), Changde City, China; 2 Department of Pathology, Changde Hospital, Xiangya School of Medicine, Central South University (The First People’s Hospital of Changde City), Changde City, China; 3 Department of Science and Education, Changde Hospital, Xiangya School of Medicine, Central South University (The First People’s Hospital of Changde City), Changde City, China

**Keywords:** anxiety, atrial fibrillation, cognitive behavioral therapy, major adverse cardiovascular events, SF-36

## Abstract

**Background:**

Atrial fibrillation (AF) and anxiety frequently co-occur, significantly impairing quality of life (QoL) and potentially worsening prognosis. However, integrated psychological management in AF care remains insufficient, with scarce evidence on combined pharmacotherapy and psychological interventions.

**Objective:**

This single-center, randomized controlled trial employed a 2 × 2 factorial design to evaluate the efficacy of combined anti-anxiety therapy [escitalopram and Cognitive Behavioral Therapy (CBT)] in 120 patients with AF and anxiety (GAD-7 ≥ 10). Participants were allocated to one of four groups: Drug Therapy, Psychological Intervention (CBT), Combination (Drug + CBT), or Control (routine care). The primary endpoint was the change in QoL (SF-36 questionnaire) at 3 and 6 months, and the secondary endpoint was the incidence of Major Adverse Cardiovascular Events (MACE).

**Results:**

A total of 117 patients completed the follow-up. At 6 months, significant improvements were observed in the Role-Physical (RP), General Health (GH), Vitality (VT), and Social Functioning (SF) domains of the SF-36 in the intervention groups compared to the control group (all *P* < 0.01). Factorial analysis of the GH score change confirmed significant main effects for both psychological intervention (*P* < 0.001) and drug therapy (*P* = 0.011), with no significant interaction, indicating additive benefits. The Combination group also demonstrated a significantly lower overall incidence of MACE at 6 months compared to the Control group (36.67% vs. 70.00%, *P* = 0.003).

**Conclusions:**

This study demonstrates that combined anti-anxiety pharmacotherapy and psychological therapy improves QoL and reduces cardiovascular risk in patients with AF and anxiety.

## Introduction

Atrial fibrillation (AF) is one of the most common clinical arrhythmias, with a rising global prevalence. AF symptoms significantly impair daily functioning and reduce patients’ quality of life (QoL). The severity and type of AF symptoms not only affect QoL but can also contribute to emotional and psychological disorders (1). Current AF management primarily focuses on physiological aspects such as anticoagulation, rhythm, and rate control, with insufficient integration of psychological interventions. Although some studies confirm that psychological interventions can alleviate AF-related symptoms (2) and improve adherence to anticoagulant therapy in AF patients with comorbid anxiety and depression (3), thereby reducing stroke-related risks, research on pharmacological interventions specifically for AF patients with anxiety remains scarce. Furthermore, an evidence-based foundation for multimodal combined interventions is lacking. Recently, a randomized controlled trial led by Professor He Huang's team at Renmin Hospital of Wuhan University highlighted the potential of Shensong Yangxin Capsule due to its dual “anti-arrhythmic and anti-anxiety” properties (4).It should be noted that our study did not administer Sheng Yangxin Capsule. Instead, we cited this clinical trial to exemplify the growing body of evidence supporting comprehensive treatment strategies for atrial fibrillation patients with comorbidities. Integrating psychological care into the comprehensive management of AF aligns with the “Psycho-Cardiology” model and provides a scientific basis for optimizing individualized treatment strategies (5). Therefore, we propose, for the first time, a factorial analysis to investigate the combined effect of pharmacological and psychological interventions in patients with AF and comorbid anxiety. This study aims to evaluate the therapeutic efficacy and assess its impact on improving medium- to long-term patient outcomes, while exploring underlying mechanisms. The findings are expected to provide novel insights and a theoretical basis for addressing psychological issues in AF, thereby advancing the development of related treatment strategies.

## Materials and methods

### Study design

This was a single-center, randomized controlled trial with a 2 × 2 factorial design.

### Study population

**Figure 1:**
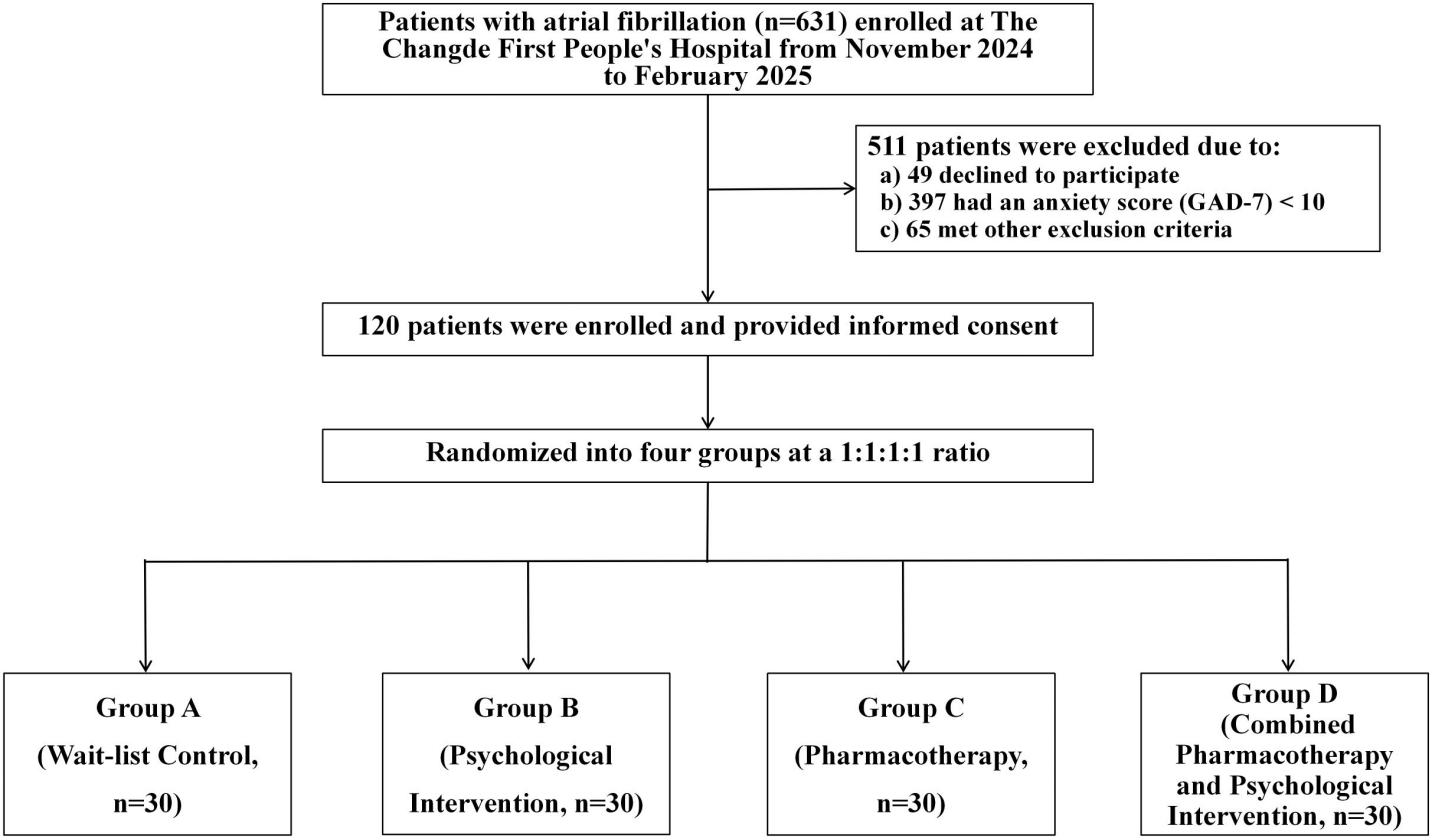
Patient screening flowchart.

According to the inclusion and exclusion criteria, patients with AF and comorbid anxiety at the First People's Hospital of Changde City were enrolled between November 1, 2024, and February 28, 2025. Ultimately, 120 patients were included in this phase of the study for analysis (Figure 1). The study was conducted in accordance with the Declaration of Helsinki and approved by the Ethics Committee of the lead unit (The First People's Hospital of Changde City; Approval No: YX-2023-440-05, Approval Date: April 20, 2024). Written informed consent was obtained from all participants.and was registered in the Chinese Medical Research Registration and Filing Information System (No. MR-43-25-021612).

### Diagnostic criteria

The 2021 European Society of Cardiology (ESC) guidelines (6) classify atrial fibrillation (AF) into four subtypes: paroxysmal, persistent, long-standing persistent, and permanent AF. The present study enrolled patients with paroxysmal, persistent, or long-standing persistent AF, all of whom had definite AF episodes confirmed by standard 12-lead electrocardiography (ECG) or dynamic Holter monitoring. The diagnostic ECG characteristics of AF included absent *P* waves, irregular fibrillatory (f) waves, and completely irregular ventricular rhythm. Patients with permanent AF were excluded from this study, considering the distinct clinical management strategies for this AF subtype.

#### Anxiety state

Based on previous research, a web-based calculator application using the AAR0T0 prediction model assessed the risk of anxiety in AF patients. Patients were defined as having an anxiety state when the Generalized Anxiety Disorder-7 (GAD-7) score was ≥ 10.

### Inclusion and exclusion criteria

#### Inclusion criteria

(1) Patients with paroxysmal, persistent, or permanent AF (diagnosed per the 2021 ESC guidelines); (2) GAD-7 score ≥ 10; (3) Age between 18 and 80 years.

#### Exclusion criteria

(1) Lack of confirmed AF evidence in medical records; (2) Comorbid severe organic diseases, such as severe respiratory failure, malignant tumors, severe hepatic or renal dysfunction, or heart failure (left ventricular ejection fraction <40% or NYHA class IV); (3) Severe arrhythmias, including sick sinus syndrome, atrioventricular block, or long QT syndrome; (4) Severe thyroid dysfunction; (5) Previous diagnosis of psychiatric disorders or cognitive dysfunction, with long-term use of antipsychotic medications; (6) Known allergy to escitalopram; (7) GAD-7 score < 10.

### Randomization and intervention

Eligible subjects were numbered sequentially based on the order of enrollment. A staff member not involved in patient recruitment generated random numbers using a random number table to create the final allocation sequence. Participants were randomly assigned in a 1:1:1:1 ratio to one of four groups: Drug Therapy Group, Drug Therapy + Psychological Intervention Group, Psychological Intervention Group, or Control Group. The allocation for each participant was concealed in the randomization table. Physicians then prescribed the corresponding intervention based on this assignment.Anti-anxiety Medication: According to guidelines, escitalopram was administered starting at 5 mg/day, titrated to 10 mg/day, and continued for 3 months.Psychological Intervention: Patients accessed the AF chronic disease management module via the hospital's platform. They received weekly Cognitive Behavioral Therapy (CBT) sessions for 3 months (12 sessions total) (7), focusing on stress management and emotional regulation. Conventional Therapy: All patients received standard care, including anticoagulants (e.g., rivaroxaban, warfarin, dabigatran, edoxaban), rhythm control agents (e.g., dronedarone, amiodarone), and ventricular rate control (e.g., metoprolol succinate sustained-release tablets).

### Outcome measures

(1) Baseline Data

Baseline Assessment: Height, weight, Body Mass Index (BMI).

Clinical Symptoms: Palpitations, chest pain, dyspnea.

Clinical History: Hypertension, coronary heart disease, diabetes, hyperlipidemia, cerebral apoplexy, drink alcohol and smoking.

Vital Signs: Resting heart rate and blood pressure.

Laboratory Tests: Complete blood count, urinalysis, liver and kidney function, electrolytes, lipid profile, blood glucose, coagulation function, PT,INR.

Echocardiography: Left and right ventricular dimensions, left and right atrial dimensions, Left Ventricular Ejection Fraction (LVEF).

Quality of Life Questionnaire: The SF-36 Health Survey was used to analyze QoL across eight domains: Physical Functioning (PF), Role-Physical (RP), Bodily Pain (BP), General Health (GH), Vitality (VT), Social Functioning (SF), Role-Emotional (RE), and Mental Health (MH).

(2) Follow-up

Method: Face-to-face visits.

Time Points: 3 and 6 months.

Parameters: Clinical symptoms, height, weight, SF-36 questionnaire; vital signs (heart rate, blood pressure); auxiliary examinations (ECG, echocardiography).

(3) Endpoints

Primary Endpoint: Quality of Life (SF-36 score).

Secondary Endpoint: Major Adverse Cardiovascular Events (MACE).

### Sample size estimation

For the 2 × 2 factorial design (8), the sample size was estimated using the simplified formula: *n* = 4 * (Z*α*/2 + Z*β*)^2^ * *σ*^2^/*Δ*^2^. Here, *n* is the total sample size; *σ*^2^ is the within-group variance; Z*α*/2 is the Z-value for the significance level (set to 2.56 for a two-sided *α*=0.01); Z*β* is the Z-value for statistical power (set to 1.28 for 90% power); and *Δ* is the effect size (set to 0.8, indicating a large effect). Assuming similar effect sizes for drug and psychological interventions with no interaction, the calculation yielded *n* = 101. Accounting for a 20% dropout rate, the final sample size was inflated to 121 patients, with 30 patients per group.

### Statistical analysis

Data were analyzed using SPSS software (version 30.0). The normality of continuous variables was assessed using the Kolmogorov–Smirnov test. Normally distributed continuous data are expressed as mea*n* ± standard deviation (SD) and were compared among multiple groups using one-way analysis of variance (ANOVA), with *post-hoc* pairwise comparisons performed using the Bonferroni correction (9). Categorical data are presented as numbers (percentages) and were compared using the chi-square test. Patients were categorized into Combination Group, Drug Therapy Group, Psychological Intervention Group, and Control Group. Comparisons between the Combination Group and the other groups were performed using t-tests and chi-square tests, including analyses for overall superiority (Combination vs. Control) and extensive superiority (Combination vs. Drug Therapy alone and vs. Psychological Intervention alone). Factorial analysis of covariance (ANCOVA) (10) was employed to examine the main effects of the two interventions (drug and psychological) and their additive interaction, with baseline SF-36 scores included as a covariate. Furthermore, the Cochran-Mantel-Haenszel (CMH) test (11) was used to control for potential heterogeneity across different centers. Time to first MACE was analysed using the Kaplan–Meier method, and between-group differences were compared using the log-rank test. A two-sided *P*-value ≤ 0.05 was considered statistically significant.

## Results

### Baseline characteristics

A total of 631 AF patients were screened during the study period. Of these, 49 declined to participate, 397 had a GAD-7 score < 10, and 65 met other exclusion criteria, leaving 120 patients who were ultimately enrolled and randomized (Figure 1).

According to the 1:1:1:1 randomization scheme, patients were allocated to: Group A (Control group, *n* = 30), Group B (Psychological Intervention group, *n* = 30), Group C (Drug Therapy group, *n* = 30), and Group D (Drug Therapy + Psychological Intervention group, *n* = 30). No significant differences were observed at baseline among the four groups regarding age, sex, daily sleep duration, occupation type, presence of palpitations, AF type, anticoagulant use, *β*-blocker use, height, heart rate, neutrophil count, fasting blood glucose, high-density lipoprotein (HDL) levels, CHA₂DS₂-VASc score, HAS-BLED score, all eight domains of the SF-36 (PF, RP, BP, GH, VT, SF, RE, MH), or length of hospital stay (all *P* > 0.05) (Table 1).

**Table 1:** Baseline characteristics of study participants.

Characteristic	Group A (*n* = 30)	Group B (*n* = 30)	Group C (*n* = 30)	Group D (*n* = 30)	*p*-value
Age (years)	71.07 ± 5.89	69.10 ± 6.77	67.70 ± 7.89	65.73 ± 9.42	0.107
Sex, *n* (%)					0.518
Female	17 (56.67)	16 (53.33)	12 (40.00)	17 (56.67)	
Male	13 (43.33)	14 (46.67)	18 (60.00)	13 (43.33)	
Daily Sleep Duration (hours), *n* (%)					0.432
≤ 6	18 (60.00)	22 (73.33)	21 (70.00)	16 (53.33)	
> 6, ≤8	11 (36.67)	6 (20.00)	9 (30.00)	13 (43.33)	
> 8	1 (3.33)	2 (6.67)	0 (0.00)	1 (3.33)	
Occupation Type, *n* (%)					0.986
Mental Labor	23 (76.67)	23 (76.67)	24 (80.00)	23 (76.67)	
Manual Labor	7 (23.33)	7 (23.33)	6 (20.00)	7 (23.33)	
Palpitations, *n* (%)					0.948
No	3 (10.00)	2 (6.67)	2 (6.67)	2 (6.67)	
Yes	27 (90.00)	28 (93.33)	28 (93.33)	28 (93.33)	
AF Type, *n* (%)					0.725
Paroxysmal	10 (33.33)	10 (33.33)	9 (30.00)	13 (43.33)	
Persistent	20 (66.67)	20 (66.67)	21 (70.00)	17 (56.67)	
Anticoagulant, *n* (%)					0.965
No	7 (23.33)	4 (13.33)	7 (23.33)	7 (23.33)	
Rivaroxaban	19 (63.33)	21 (70.00)	19 (63.33)	17 (56.67)	
Warfarin	2 (6.67)	3 (10.00)	3 (10.00)	4 (13.33)	
Dabigatran	1 (3.33)	1 (3.33)	1 (3.33)	2 (6.67)	
Edoxaban	1 (3.33)	1 (3.33)	0 (0.00)	0 (0.00)	
Height (m)	1.64 ± 0.08	1.62 ± 0.09	1.63 ± 0.08	1.61 ± 0.09	0.747
Heart Rate (beats/min)	81.40 ± 17.21	87.40 ± 21.30	81.73 ± 24.84	80.37 ± 19.58	0.442
Neutrophils (×10⁹/L)	4.37 ± 1.44	4.77 ± 2.89	4.48 ± 1.84	4.51 ± 2.37	0.925
Fasting Blood Glucose (mmol/L)	5.90 ± 1.79	5.90 ± 2.56	6.32 ± 3.23	5.54 ± 1.87	0.678
HDL-C (mmol/L)	1.00 ± 0.25	1.02 ± 0.30	1.00 ± 0.33	1.00 ± 0.24	0.936
CHA₂DS₂-VASc Score	3.77 ± 1.43	3.67 ± 1.58	3.20 ± 1.77	3.20 ± 1.71	0.304
HAS-BLED Score	2.20 ± 1.16	2.07 ± 1.17	1.57 ± 1.17	1.80 ± 1.10	0.145
SF-36 Scores					
Physical Functioning (PF)	30.00 ± 5.72	29.50 ± 5.78	29.83 ± 5.49	29.83 ± 6.09	0.990
Role-Physical (RP)	17.50 ± 17.56	22.50 ± 16.54	19.17 ± 18.20	22.50 ± 18.97	0.600
Bodily Pain (BP)	48.13 ± 8.82	46.93 ± 8.41	45.83 ± 8.19	46.80 ± 8.52	0.323
General Health (GH)	35.97 ± 8.04	35.20 ± 7.90	34.63 ± 7.77	36.00 ± 7.75	0.864
Vitality (VT)	63.17 ± 5.49	61.67 ± 4.79	63.33 ± 6.21	64.50 ± 6.99	0.455
Social Functioning (SF)	47.08 ± 10.22	45.83 ± 11.05	48.61 ± 10.01	47.50 ± 9.51	0.782
Role-Emotional (RE)	15.55 ± 20.96	14.43 ± 16.78	15.54 ± 16.90	19.99 ± 22.49	0.816
Mental Health (MH)	44.27 ± 5.35	45.07 ± 4.45	45.20 ± 4.60	44.67 ± 3.94	0.912
Length of Hospital Stay (days)	7.13 ± 2.43	6.73 ± 2.48	6.70 ± 2.88	6.83 ± 2.60	0.884

*Indicates a statistically significant difference (*p* < 0.05).

### Changes in SF-36 scores across groups

**Figure 2:**
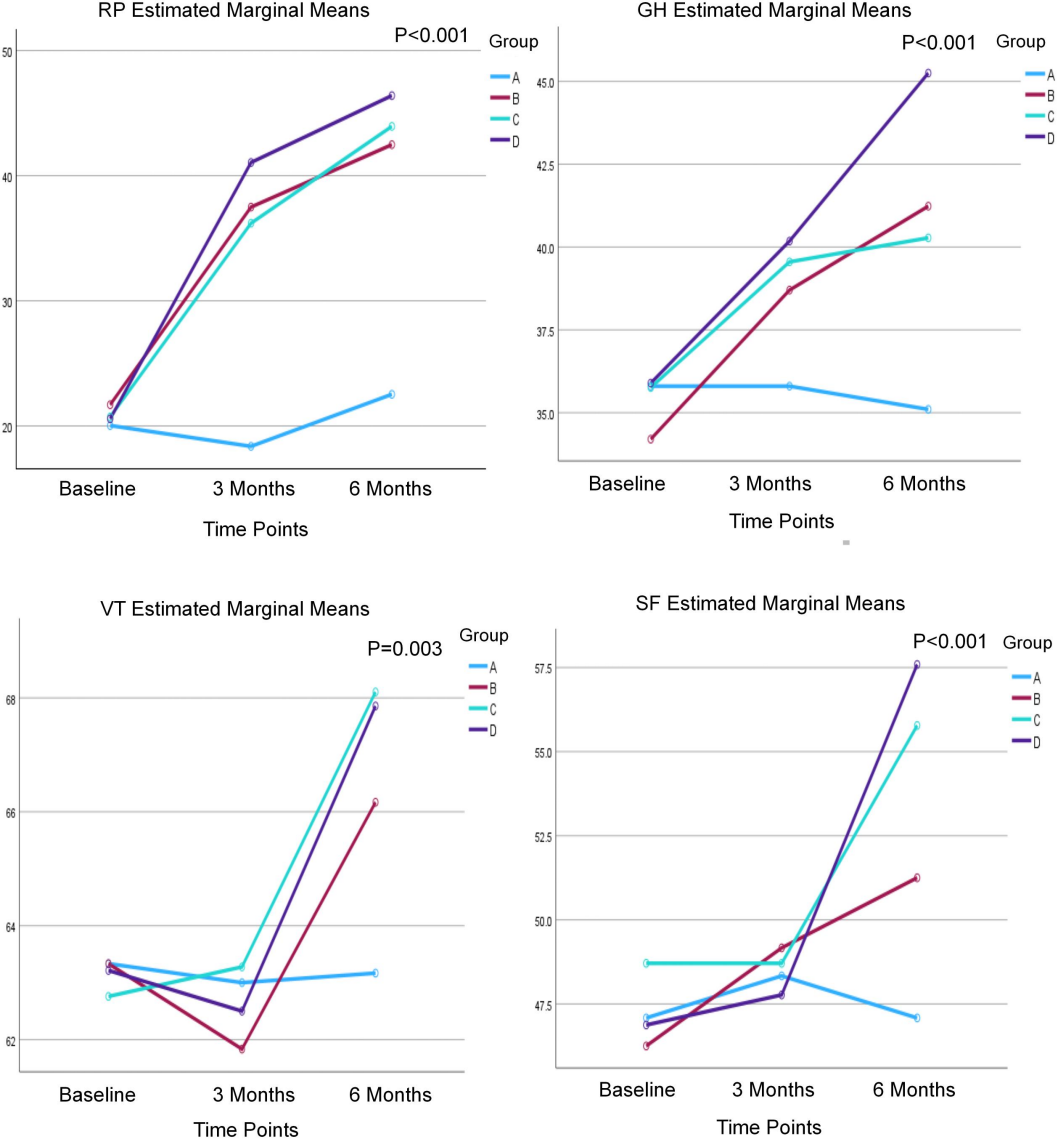
Changes in estimated marginal means for four subscales of the SF-36 across the study period.

Among the 120 randomized patients, one in Group C and two in Group D withdrew due to unwillingness to continue escitalopram during the 3-month treatment period. Consequently, 117 patients completed the follow-up (Group A: *n* = 30; Group B: *n* = 30; Group C: *n* = 29; Group D: *n* = 28). No significant differences were found in the baseline scores for any SF-36 domains (PF, RP, BP, GH, VT, SF, RE, MH) among the groups (all *P* > 0.05). At the 3-month follow-up, a statistically significant difference was observed among groups for the RP domain (F = 15.824, *P* < 0.001), but not for the other domains (PF, BP, GH, VT, SF, RE, MH; all *P* > 0.05). At the 6-month follow-up, significant inter-group differences were found for the RP, GH, VT, and SF domains (F = 13.065, *P* < 0.001; F = 10.250, *P* < 0.001; F = 5.555, *P* = 0.003; F = 7.910, *P* < 0.001, respectively) (Figure 2). Differences for the PF, RE, and MH domains remained non-significant (all *P* > 0.05) (Table 2).

**Table 2:** Comparison of SF-36 scores across different groups at baseline, 3 months, and 6 months.

SF-36 Scale	Time point	Group A (*n* = 30)	Group B (*n* = 30)	Group C (*n* = 29)	Group D (*n* = 28)	F-value	*p*-value
Physical Functioning (PF)							
	Baseline	30.00 ± 5.72	29.50 ± 5.78	30.00 ± 5.51	29.46 ± 5.98	0.079	0.975
	3 Months	31.00 ± 4.81	34.00 ± 5.63	34.31 ± 5.46	32.68 ± 5.52	2.349	0.102
	6 Months	30.83 ± 5.43	32.33 ± 4.30	33.79 ± 5.61	33.57 ± 5.25	2.041	0.100
Role-Physical (RP)							
	Baseline	20.00 ± 17.86	21.67 ± 18.26	20.69 ± 17.76	20.54 ± 18.07	0.045	0.988
	3 Months	18.33 ± 13.02	37.50 ± 14.31	36.21 ± 12.65	41.07 ± 15.54	15.824	<0.001*
	6 Months	22.50 ± 15.19	42.50 ± 16.28	43.97 ± 17.24	46.43 ± 17.63	13.065	<0.001*
Bodily Pain (BP)							
	Baseline	47.60 ± 8.79	45.63 ± 7.95	47.45 ± 8.42	47.68 ± 8.99	0.388	0.801
	3 Months	46.53 ± 7.94	47.37 ± 8.76	47.45 ± 8.91	47.32 ± 8.59	0.072	0.997
	6 Months	47.20 ± 8.38	47.07 ± 8.99	48.90 ± 9.38	48.29 ± 8.89	0.286	0.852
General Health (GH)							
	Baseline	35.80 ± 8.32	34.20 ± 7.25	35.76 ± 7.94	35.76 ± 7.94	0.313	0.816
	3 Months	35.80 ± 8.32	38.70 ± 6.81	39.55 ± 7.82	40.18 ± 7.13	1.934	0.158
	6 Months	35.10 ± 8.57	41.23 ± 6.76	40.28 ± 6.13	45.25 ± 6.35	10.250	<0.001*
Vitality (VT)							
	Baseline	63.33 ± 6.06	63.33 ± 6.06	62.76 ± 5.44	63.21 ± 6.12	0.062	0.986
	3 Months	63.00 ± 5.66	61.83 ± 5.33	63.28 ± 5.87	62.50 ± 4.81	0.402	0.763
	6 Months	63.17 ± 5.65	66.17 ± 4.86	68.10 ± 5.58	67.86 ± 4.80	5.555	0.003*
Social Functioning (SF)							
	Baseline	47.08 ± 10.22	46.25 ± 10.46	48.71 ± 9.65	46.88 ± 10.55	0.184	0.835
	3 Months	48.33 ± 9.70	49.17 ± 10.35	48.71 ± 9.65	47.77 ± 9.04	0.249	0.896
	6 Months	47.08 ± 10.22	51.25 ± 8.27	55.78 ± 9.07	57.59 ± 8.57	7.910	<0.001*
Role-Emotional (RE)							
	Baseline	18.88 ± 22.63	17.77 ± 20.96	13.78 ± 16.69	13.08 ± 16.56	0.637	0.775
	3 Months	19.99 ± 20.71	21.09 ± 18.53	17.22 ± 16.93	19.03 ± 16.78	0.235	0.904
	6 Months	23.32 ± 23.41	26.65 ± 20.34	20.67 ± 16.44	28.55 ± 17.48	0.910	0.416
Mental Health (MH)							
	Baseline	44.67 ± 4.59	44.76 ± 4.58	44.55 ± 4.75	45.14 ± 4.60	0.091	0.944
	3 Months	45.20 ± 4.22	45.07 ± 4.45	45.24 ± 3.56	46.00 ± 4.15	0.300	0.822
	6 Months	45.47 ± 4.13	45.60 ± 4.28	46.07 ± 3.14	46.57 ± 3.48	0.503	0.649

Data are presented as mean ± standard deviation. PF, physical functioning; RP, role-physical; BP, bodily pain; GH, general health; VT, vitality; SF, social functioning; RE, role-emotional; MH, mental health.

* Indicates a statistically significant difference (*p* < 0.05).

### Changes in RP, GH, VT, and SF scores: combination vs. Single Intervention Groups

Patients were re-categorized into Combination group (Drug + Psychological), Drug Therapy group, and Psychological Intervention group to compare the changes (*Δ*) in RP, GH, VT, and SF scores from baseline at 3 and 6 months. At the 3-month mark, no significant differences were observed in the change scores for RP, GH, VT, or SF among these three groups (all *P* > 0.05). However, at 6 months, a statistically significant difference was found for the change in GH scores (F = 3.191, *P* = 0.046). The changes in RP, VT, and SF scores remained not significantly different among the groups (all *P* > 0.05) (Table 3).

**Table 3:** Superiority comparison of the combined intervention group vs. Monotherapy Groups on Changes in RP, GH, VT, and SF Scores.

SF-36 Scale	Time point	Group B (*n* = 30)	Group C (*n* = 29)	Group D (*n* = 28)	F-value	*p*-value
Role-Physical (RP)						
	3 Months	15.83 ± 12.25	15.52 ± 21.56	20.54 ± 22.62	0.605	0.548
	6 Months	20.83 ± 9.48	23.28 ± 24.94	25.89 ± 19.82	0.509	0.603
General Health (GH)						
	3 Months	4.67 ± 1.83	3.45 ± 2.71	4.29 ± 1.78	2.470	0.091
	6 Months	7.03 ± 8.00	4.52 ± 7.06	9.36 ± 6.51	3.191	0.046*
Vitality (VT)						
	3 Months	−1.50 ± 6.18	0.52 ± 6.99	−0.71 ± 8.47	0.579	0.563
	6 Months	2.83 ± 5.83	5.34 ± 6.67	4.64 ± 8.81	0.964	0.386
Social Functioning (SF)						
	3 Months	2.92 ± 12.58	0.00 ± 13.36	0.89 ± 13.58	0.119	0.685
	6 Months	5.00 ± 12.54	7.07 ± 12.82	10.71 ± 12.13	1.541	0.220

Data are presented as mean change from baseline ± standard deviation. RP, role-physical; GH, general health; VT, vitality; SF, social functioning.

* Indicates a statistically significant difference (*p* < 0.05).

### Factorial analysis of GH score changes at 6 months

**Figure 3:**
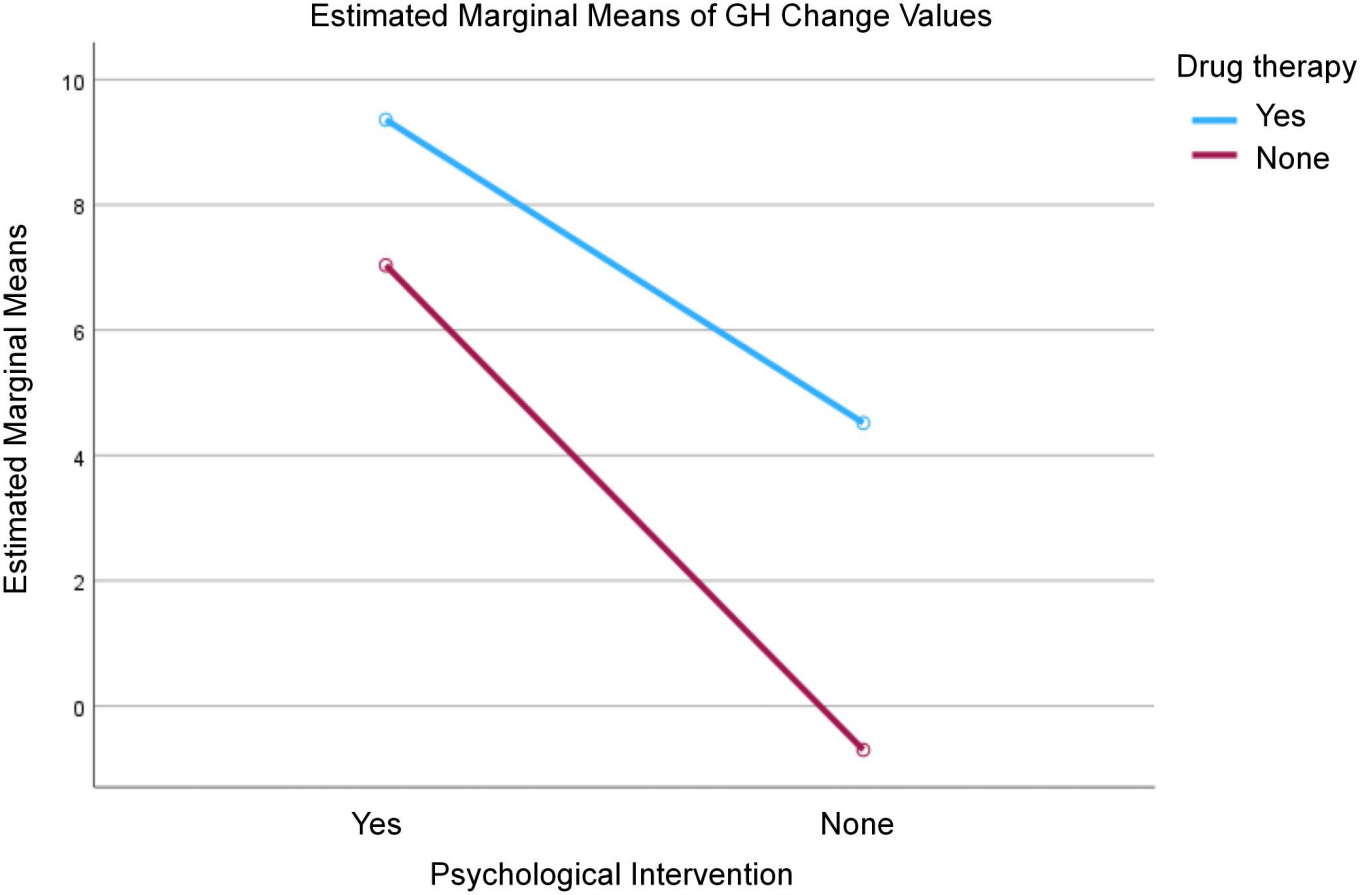
Estimated marginal means of GH change values by psychological intervention.

A 2 × 2 factorial analysis was performed with the change in GH score at 6 months as the outcome variable, and drug therapy and psychological intervention as the two factors. Levene's Test for equality of variances was non-significant (*P* = 0.582) (Table 4), confirming homogeneity of variances. The subsequent tests of between-subjects effects revealed that both psychological intervention and drug therapy significantly increased the GH change score (*P* < 0.001 and *P* = 0.011, respectively) (Table 5). However, no significant interaction effect was found between the two interventions (Figure 3).

**Table 4:** Levene’s test for homogeneity of variances (dependent Variable: change in GH score).

	Levene Statistic	df1	df2	Significance
Based on Mean	0.654	3	113	0.582
Based on Median	0.452	3	113	0.717
Based on Median and with adjusted df	0.452	3	96.693	0.717
Based on trimmed mean	0.680	3	113	0.566

Design: Intercept + Psychological Intervention + Pharmacotherapy + Psychological Intervention * Pharmacotherapy.

Dependent Variable: Change in GH Score.

R-Squared = 0.189 (Adjusted R-Squared = 0.167).

**Table 5:** Tests of between-subjects effects.

Source	Type III Sum of Squares	df	Mean Square	F	Significance
Corrected Model	1637.029a	3	545.676	8.775	<0.001*
Intercept	2983.639	1	2983.639	47.980	<0.001*
Psychological Intervention	1155.063	1	1155.063	18.575	<0.001*
Pharmacotherapy	415.504	1	415.504	6.682	0.011*
Psychological Intervention* Pharmacotherapy	61.170	1	61.170	0.984	0.323
Error	7026.937	113	62.185		
Total	11569.000	117			
Corrected Total	8663.966	116			

Dependent Variable: Change in GH Score.

*Indicates a statistically significant difference (*p* < 0.05).

### Comparison of MACE at 3 and 6 months

**Figure 4:**
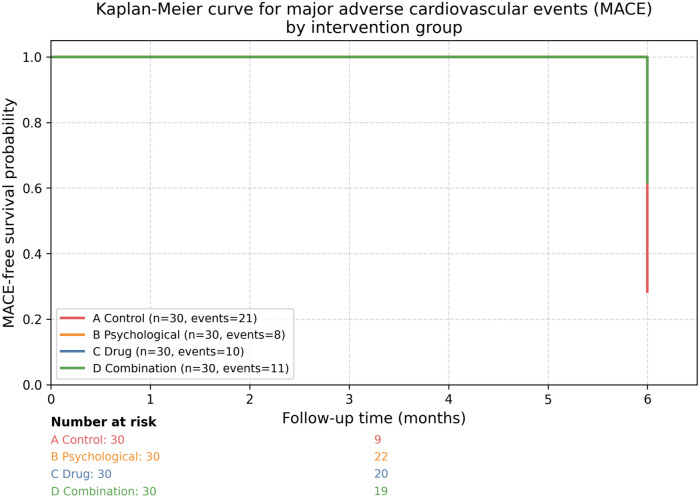
The Kaplan –Meier curve for MACE.

At the 3-month follow-up, there was no significant difference in the overall incidence of adverse events among Groups A, B, C, and D (*P* = 0.323). Furthermore, the incidence rates of individual events—recurrent palpitations, heart failure, angina, stroke, bleeding events (one case of gastrointestinal bleeding in Group A), and rehospitalization—did not differ significantly among the groups (Table 6). By the 6-month follow-up, a statistically significant difference emerged in the overall incidence of adverse events among the groups (*P* = 0.003) (Refer to summary table). However, for the specific individual events—recurrent palpitations, heart failure, angina, stroke (one cerebral infarction each in Groups A and C), bleeding events (one gastrointestinal bleeding each in Groups A and D), and rehospitalization—the differences in incidence rates were not statistically significant (Table 6). The Kaplan–Meier curve for MACE-free survival is shown in Figure 4.

**Table 6:** Adverse events at 3 and 6 months [n (%)].

Time Point	Group	Adverse Events (Overall)	Palpitation	Heart Failure	Angina Pectoris	Stroke	Bleeding	Rehospitalization
3 Months	A	18 (60.00%)	11 (36.67%)	5 (16.67%)	9 (30.00%)	0 (0.00%)	1 (3.33%)	4 (13.33%)
	B	13 (43.33%)	5 (16.67%)	4 (13.33%)	8 (26.67%)	0 (0.00%)	0 (0.00%)	3 (10.00%)
	C	14 (46.67%)	7 (23.33%)	5 (16.67%)	6 (20.00%)	0 (0.00%)	0 (0.00%)	5 (16.67%)
	D	11 (36.67%)	5 (16.67%)	3 (10.00%)	5 (16.67%)	0 (0.00%)	0 (0.00%)	3 (10.00%)
	*p*-value	0.323	0.215	0.860	0.601	NA	0.412	0.902
6 Months	A	21 (70.00%)	10 (33.33%)	10 (33.33%)	5 (16.67%)	1 (3.33%)	1 (3.33%)	13 (43.33%)
	B	8 (26.67%)	5 (16.67%)	5 (16.67%)	1 (3.33%)	0 (0.00%)	0 (0.00%)	6 (20.00%)
	C	10 (33.33%)	5 (16.67%)	4 (13.33%)	4 (13.33%)	1 (3.33%)	0 (0.00%)	8 (26.67%)
	D	11 (36.67%)	4 (13.33%)	3 (10.00%)	3 (10.00%)	0 (0.00%)	1 (3.33%)	6 (20.00%)
	*p*-value	0.003	0.205	0.091	0.389	0.565	0.419	0.140

## Discussion

This 2 × 2 factorial randomized controlled trial is the first to systematically investigate the effects of combined anti-anxiety pharmacotherapy and psychological intervention on outcomes in patients with AF and comorbid anxiety. The results demonstrated that the drug therapy, psychological intervention, and combination groups showed significantly greater improvements in GH, RP, VT, and SF scores compared to the control group. Factorial analysis of the change in GH score at 6 months confirmed that both psychological intervention and drug therapy independently and significantly increased the GH change value. Furthermore, the combination group exhibited a reduction in the overall incidence of MACE at the 6-month follow-up. These findings provide important evidence for the integrated management of AF patients with anxiety and support the clinical application of the “Psycho-Cardiology” model.

### Synergistic effects of combined intervention

The factorial design elucidated the independent and combined effects of drug therapy and psychological intervention. The results showed a significant main effect for psychological intervention (*P* < 0.001) and for drug therapy (*P* = 0.011) on GH improvement, but no significant interaction was observed (*P* = 0.323). This indicates that the combined application of drug and psychological interventions improves patients’ general health status through independent, additive pathways. For instance, drug therapy may directly modulate the balance of the serotonin (5-HT) system, which plays a key role in the neuro-cardiovascular interplay in AF patients (12). 5-HT, a monoamine neurotransmitter widely distributed in the central nervous system and peripheral tissues (e.g., platelets, gut), has receptors (e.g., 5-HT1A, 5-HT2A, 5-HT3) importantly expressed in cardiac autonomic regulation, inflammatory responses, and endothelial function (13). Conversely, psychological intervention, via CBT, alleviates anxiety, and the two approaches synergistically reduce the psychological burden in AF patients, thereby indirectly improving cardiac function (14). In this study, the combination group showed a significantly greater improvement in GH at 6 months compared to the single intervention groups (*P* = 0.046), and a statistically significant difference in the overall incidence of adverse events among groups emerged at 6 months (*P* = 0.003), further underscoring the importance of comprehensive management in long-term intervention.

### Multidimensional assessment of quality of life

The SF-36 questionnaire is an important instrument for assessing outcomes in AF patients (15). This study found particularly prominent improvements in the RP, GH, VT, and SF domains within the combination group. For example, the SF score in the combination group increased by 10.71 points from baseline at 6 months, significantly higher than in other groups (*P* < 0.001). This might be related to enhanced social participation and self-efficacy fostered by the psychological intervention. Previous research suggests that psychological interventions can improve emotional regulation, thereby reducing social avoidance behaviors and enhancing social functioning in AF patients (16). The improvement in VT scores (increase of 4.64 points in the combination group) might reflect the alleviation of fatigue symptoms by the anti-anxiety medication. Escitalopram, for instance, may modulate norepinephrine levels, leading to indirect anti-inflammatory, anti-ischemic, antiarrhythmic, and antithrombotic effects, potentially reducing AF risk factors (17) and improving vitality. General Health is a significant indicator of prognosis in AF patients, with multiple studies suggesting that changes in GH scores have good reliability and validity for assessing QoL in hospitalized AF patients (18, 19).

### Adverse cardiovascular events in AF patients

Major Adverse Cardiovascular Events (MACE) serve as a composite clinical endpoint for evaluating prognosis or treatment efficacy in cardiovascular disease patients. Its application is common in prognostic studies of coronary artery disease (CAD) (20, 21) or AF (16, 22). MACE typically includes heart failure, recurrent angina, non-fatal re-infarction, thromboembolic events, cardiovascular-related rehospitalization, and cardiac death (23, 24). In AF-specific studies, however, the short-term incidence of events like non-fatal re-infarction, thromboembolism, and cardiac death is often low. Considering the low expected probability of traditional MACE in this study population and referencing other literature, we defined the secondary endpoint as adverse cardiovascular events or an expanded MACE definition, including rehospitalization due to recurrent palpitations, heart failure, recurrent chest pain, or newly developed thromboembolic and bleeding events (25, 26). Although the incidence rates of individual adverse events (e.g., stroke, bleeding, recurrent chest pain, heart failure, recurrent palpitations) did not differ significantly between groups, the overall incidence of adverse cardiovascular events in the combination group was significantly lower than in the control group at 6 months (36.67% vs. 70.00%, *P* = 0.003).

### Application of anti-anxiety therapy in AF patients

Autonomic nervous system imbalance is a key mechanism linking psychological state and cardiac pathology in AF patients with anxiety (27). Anxiety not only increases symptom burden but may also promote AF recurrence through mechanisms like sympathetic activation and inflammatory responses (28). Consequently, anti-anxiety treatment is increasingly recognized as a crucial component of comprehensive AF management. Selective serotonin reuptake inhibitors (SSRIs) and serotonin-norepinephrine reuptake inhibitors (SNRIs) remain the mainstay pharmacological treatments (29). A recent systematic review found that psychological interventions improved depression, anxiety, and mental HRQoL, with the most pronounced effects observed for anxiety interventions. The increasing use of multicomponent psychological interventions suggests a promising approach to incorporating patients’ needs and preferences. Further investigations are warranted in patients at high risk of poor prognosis to compare intervention components and outcomes in AF patients (30). Different classes of psychotropic medications have distinct profiles:SSRIs (e.g., sertraline, citalopram, fluoxetine): Advantages include high cardiovascular safety (minimal effects on heart rate, blood pressure), making them suitable for patients with hypertension or CAD. Studies like SADHART (31) recommend sertraline and citalopram as first-line agents for CVD with depression, noting fewer drug interactions and lower risks with beta-blockers/anticoagulants (though fluoxetine may potentiate warfarin).SNRIs (e.g., venlafaxine, duloxetine): Advantages include potent antidepressant effects, beneficial for treatment-resistant depression or somatic symptoms. Disadvantages include potential elevation of blood pressure and heart rate (especially venlafaxine >150 mg/day), contraindicating use in uncontrolled hypertension or heart failure.Tricyclic Antidepressants (TCAs, e.g., amitriptyline, imipramine): Advantages include potent anxiolytic/antidepressant effects, useful for insomnia/pain. Major disadvantages are QT prolongation and increased risk of ventricular arrhythmias (32).Other Novel Antidepressants (e.g., mirtazapine): Advantages include improved sleep/appetite, suitable for patients with weight loss or insomnia. Disadvantages include potential weight gain and metabolic syndrome (33).Benzodiazepines (e.g., lorazepam, diazepam): Advantages include rapid onset, suitable for preoperative anxiety or acute panic attacks post-MI. Disadvantages include risks of hypotension, respiratory depression (worsening CHF/COPD), falls, cognitive impairment with long-term use (caution in elderly), and potential for dependence (34).After extensive discussion with psychiatrists and the hospital ethics committee, escitalopram was selected for this arrhythmia patient study to avoid dependency issues and potential risks associated with higher citalopram doses. During our mid-term follow-up, aside from three patients who discontinued escitalopram due to perceived symptom resolution, no other severe adverse drug reactions were reported.

### Optimized psychological intervention pathways in AF patients

CBT is a problem-oriented, structured psychotherapy that improves emotional and psychological issues by modifying cognitions and behaviors (35). Regarding CBT in cardiovascular diseases, recent domestic literature reports indicate that CBT can reduce MACE in heart failure and other cardiovascular disease patients (36, 37). Literature on CBT application in AF is also emerging (2, 14), generally concluding that CBT effectively improves QoL and alleviates psychological distress in AF patients. This study utilized the hospital's chronic disease management platform for regular patient contact and CBT sessions (once weekly, 12 sessions total) delivered by dedicated personnel. While our results regarding psychological outcomes might not be as pronounced as some previous studies, the significant reduction in overall adverse cardiovascular events in the combination group at 6 months highlights its clinical value. Current AF management guidelines primarily focus on anticoagulation, rhythm control, and risk factor management, with insufficient integration of psychological interventions. This factorial design study confirms that combined intervention is superior to single-modality approaches in reducing cardiovascular risk, aligning with the “Psycho-Cardiology” model and offering a new direction for personalized AF treatment. Future large-scale, long-term studies are needed to validate these long-term benefits and explore pathology-based precision intervention strategies.

### Limitations

This study has several key limitations. First, each group had a limited sample size of 30 participants, which reduced the statistical power for subgroup analyses. The 6-month follow-up period was relatively short to capture long-term clinical outcomes such as stroke and mortality, and continuous arrhythmia monitoring was not performed to evaluate AF recurrence. Second, anxiety was assessed solely using the GAD-7 scale without cross-validation with other standardized tools, potentially introducing measurement bias. Third, the single-center design and the lack of a unified drug titration protocol may compromise the generalizability of our findings. In addition, unmeasured lifestyle factors, including yoga, meditation, weight management and substance use, were not systematically collected or adjusted for, which may interfere with anxiety status and AF recurrence and confound the observed effects of CBT. Finally, potential confounding interactions between baseline *β*-blocker exposure and psychological outcomes cannot be fully excluded. The open-label design and the self-reported nature of the SF-36 primary endpoint introduce risks of performance and detection bias. Although MACE endpoints were adjudicated by blinded assessors and statistical analysts were masked to group allocation, we cannot fully exclude the influence of participant expectations on QoL outcomes.

## Conclusion

This study demonstrates that combined anti-anxiety pharmacotherapy and psychological therapy improves QoL and reduces cardiovascular risk in patients with AF and anxiety.

## Data Availability

The original contributions presented in the study are included in the article/Supplementary Material, further inquiries can be directed to the corresponding author.
